# *In silico* Characterization of Human Prion-Like Proteins: Beyond Neurological Diseases

**DOI:** 10.3389/fphys.2019.00314

**Published:** 2019-03-27

**Authors:** Valentin Iglesias, Lisanna Paladin, Teresa Juan-Blanco, Irantzu Pallarès, Patrick Aloy, Silvio C. E. Tosatto, Salvador Ventura

**Affiliations:** ^1^Institut de Biotecnologia i de Biomedicina, Departament de Bioquímica i Biologia Molecular, Universitat Autònoma de Barcelona, Barcelona, Spain; ^2^Department of Biomedical Sciences, University of Padua, Padua, Italy; ^3^Joint IRB-BSC-CRG Program in Computational Biology, Institute for Research in Biomedicine (IRB Barcelona), The Barcelona Institute of Science and Technology, Barcelona, Spain; ^4^Institució Catalana de Recerca i Estudis Avançats, Barcelona, Spain; ^5^CNR Institute of Neuroscience, Padua, Italy

**Keywords:** prion-like proteins, disease, protein–protein interaction, protein aggregation, amyloid, bioinformatics

## Abstract

Prion-like behavior has been in the spotlight since it was first associated with the onset of mammalian neurodegenerative diseases. However, a growing body of evidence suggests that this mechanism could be behind the regulation of processes such as transcription and translation in multiple species. Here, we perform a stringent computational survey to identify prion-like proteins in the human proteome. We detected 242 candidate polypeptides and computationally assessed their function, protein–protein interaction networks, tissular expression, and their link to disease. Human prion-like proteins constitute a subset of modular polypeptides broadly expressed across different cell types and tissues, significantly associated with disease, embedded in highly connected interaction networks, and involved in the flow of genetic information in the cell. Our analysis suggests that these proteins might play a relevant role not only in neurological disorders, but also in different types of cancer and viral infections.

## Introduction

Prions were first reported in the context of mammalian neurodegenerative disorders (Prusiner, 1982; van Rheede et al., 2003; Harrison et al., 2010; Sikorska and Liberski, 2012), but it is now clear that different organisms exploit prion conformational conversion for functional purposes (Halfmann and Lindquist, 2010). The most studied organism is *Saccharomyces cerevisiae*, with up to 11 functional prions identified so far (Cascarina and Ross, 2014; Batlle et al., 2017c) Initially, these yeast prions were proposed to be pathological agents (Nakayashiki et al., 2005; McGlinchey et al., 2011), but nowadays they are widely recognized to provide beneficial advantages in changing environments, predominantly by regulating transcription, translation, or RNA processing (Halfmann et al., 2012; Newby and Lindquist, 2013). Yeast prions switch from an initially soluble state through a structural conversion toward an aggregated amyloid conformation. This conversion is encoded in PrDs; long intrinsically disordered regions of low complexity.

A significant number of proteins sharing most, but not all, prion characteristics have been identified in different organisms, and generically named prion-like proteins (Pallares et al., 2015; Si, 2015; Chakrabortee et al., 2016). In higher eukaryotes, prion-like structural conversion plays a central role in diverse functions such as viral response (Hou et al., 2011; Franklin et al., 2014; Xu et al., 2014) or long-term memory acquisition and maintenance (Si et al., 2010; Majumdar et al., 2012; Si and Kandel, 2016). Even though multiple beneficial functions have been assigned to prion-like mechanisms across all kingdoms of life, aggregated proteins in human neurodegenerative diseases such as Alzheimer’s and Parkinson’s diseases and amyotophic lateral sclerosis also share certain prion-like properties (Aguzzi and Rajendran, 2009; Gitler and Shorter, 2011; Luk et al., 2012; Stohr et al., 2012; Kim et al., 2013; Nomura et al., 2014).

The accumulated knowledge on the determinants of yeast prions conformational conversion has provided strong stimuli for the development of bioinformatics tools to uncover new PrLDs in other organisms (Michelitsch and Weissman, 2000; Harrison and Gerstein, 2003; Toombs et al., 2012; Espinosa Angarica et al., 2014; Lancaster et al., 2014; Afsar Minhas et al., 2017; Batlle et al., 2017c). Previous screenings for PrLDs in the human proteome have targeted the characteristic compositional bias of these protein regions (An and Harrison, 2016). We have recently proposed that, in addition to a distinctive amino acidic composition, PrLDs contain soft amyloidogenic sequence stretches that would contribute to trigger the initial protein self-assembly reaction (Sabate et al., 2015a,b). These cryptic amyloids were not only shown to be present and promote conformational conversion in *bona fide* yeast prions (Sant’Anna et al., 2016), but they also exist in human prion-like proteins (Batlle et al., 2017a) and appear to play key role in the induction, propagation, and inheritance of the prion state in the mammalian cytosol (Duernberger et al., 2018). The amyloid stretches embedded within PrLDs can be identified computationally (Sabate et al., 2015b; Zambrano et al., 2015).

Here we applied to the human proteome the same prediction scheme that allowed us to uncover the first *bona fide* prion-like protein in a bacterial proteome (Pallares et al., 2015; Yuan and Hochschild, 2017). Human proteins were first analyzed for the presence of regions with compositional similitude to yeast PrDs using the prion-like compositional bias (PLAAC) algorithm (Alberti et al., 2009; Lancaster et al., 2014) and afterward these protein domains were individually screened for the presence of soft amyloidogenic sequences using the pWALTZ program (Sabate et al., 2015b). Indeed, we have recently shown that such a combination of compositional and sequential PrLDs prediction provides the best accuracy when forecasting the aggregation propensities of individual human prion-like proteins (Batlle et al., 2017b).

In the present work, we computationally characterized the function, location, expression, PPI networks, and the connection to disease of the human prion-like subproteome. The picture that emerges from this analysis is that prion-like proteins are widespread expressed proteins that function in biological processes tightly associated to disease.

## Materials and Methods

### Data Acquisition

The human reference proteome dataset was obtained from Uniprot (UniProt Consortium, 2015) (Proteome ID UP000005640; release 2016_09) and scanned for PrLDs with PLAAC using as background probability the frequency of human proteome. From the initial 70,940 proteins in the proteome, 431 PrLD containing candidates were identified. Their predicted PrLDs were further evaluated with pWALTZ applying a cutoff of 60.00, as in Batlle et al. (2017a), which resulted in 242 final positive predictions (Supplementary Table S1).

### Prion-Like Domain Localization Within the Protein Sequence

Each prion-like protein sequence was divided into three segments, the N- and C- terminal, accounted for 25% of the residues each, whereas the resting 50% of the sequence was considered as internal. Each predicted PrLD was located in the sequence and the number of residues mapping in each of the segments counted.

### Functional Annotation

The GO annotation of all proteins in the prion-like dataset were collected, excluding the terms Inferred from Electronic Annotation (IEA) and filtering through the Generic GO slim developed by GO Consortium (Gene Ontology Consortium, 2015). All UniProt human proteins were used as background set to infer enrichment. A Fisher’s exact test of GO term distributions was performed in the three ontologies separately, to calculate the enrichment/depletion of dataset proteins with respect to the whole UniProt. The Bonferroni correction was applied in performing all the tests. The results are shown in Figure 3 applying the formula:

$$E=\log\frac{\text{GO freq. in }P_{PR}}{\text{Tot GO in }P_{PR}}-\log\frac{\text{GO freq. in }P_{Back}}{\text{Tot GO in }P_{Back}}$$

where GO is the GO term, P_PR_ and P_Back_ are the datasets of prion-like proteins and the whole proteome, respectively. The abbreviations freq. and Tot stay for frequency and total.

### Pfam Domains

Pfam (Finn et al., 2016) domains annotation in the dataset proteins were collected and compared to the human proteome (from UniProt). Fisher’s exact test was used to assess significance.

### Tissue and Cellular Localization

Tissue and cellular localization data of human proteins were retrieved from Human Protein Atlas (Uhlen et al., 2015). The prion-like proteins identifiers were converted to Ensembl Gene Ids. Human Protein Atlas reports a textual ranking of protein expression of each coding gene. This ranking (“none,” “low,” “medium,” “high”) was converted to numerical expressions, from 0 to 3, and each gene value for each particular tissue was collected. The expression of the complete gene set for the tissue was then averaged.

### Association to Diseases

OMIM disease annotation was extracted from the field “diseases” of the UniProt description (Amberger et al., 2015). All information regarding the associated diseases was collected from the OMIM FTP site. DisGeNET data were retrieved from DisGeNET download section (Pinero et al., 2015). For both databases, the number of proteins associated to at least one disease ID was divided by the total number of proteins, obtaining the fraction of disease-associated proteins. The results were compared to 100 random sampling of sets with the same number of proteins than the one in the database.

### Human Network Analysis

The human prion-like protein dataset was curated for duplicities and scanned for PPIs with Interactome3D (2017_06 version) (Mosca et al., 2013). Out of the 121 unique identities, 100 had annotated physical binary interactions. The degree and the number of interactions between prion-like proteins were analyzed and compared to a random distribution by sampling the complete human binary interactome in Interactome3D. Moreover, the sizes of the LCC and the MSD were measured (Menche et al., 2015). The subnetwork of prion-like proteins and their interactors were functionally characterized with DAVID database (Huang da et al., 2009) for GO and KEGG pathways enrichment (*n* = 1542). The significance of the differences was assessed by Wilcox *p*-value or empirical *p*-value.

## Results

### Human Prion-Like Proteins Prevalence and Modularity

A combination of prion-like compositional bias (PLAAC) and sequential amyloid propensity (pWALTZ) analysis was applied to the complete human proteome. This resulted in the identification of a total of 242 polypeptides (unique UniProt entries) bearing PrLDs (Supplementary Table S1). Our list of candidates included all human prion-like proteins shown to behave as such both *in vitro* and *in vivo*: FUS (Ju et al., 2011), TDP-43 (Wang et al., 2012), EWS (Couthouis et al., 2012), hnRNP A1 and hnRNP A2 (Kim et al., 2013), TIA1 (Li et al., 2014), and TAF15 (Couthouis et al., 2011) proteins, reinforcing the suitability of our dataset for the further evaluation of the global properties of human prion-like sequences.

According to our predictions, prion-like proteins account for a 0.34% of the human proteome. This is in line with two previous independent surveys for human prion-like proteins that exploited compositional bias alone for their detection; both studies predicting that the prevalence of these proteins is <1% (An and Harrison, 2016). Despite the percentage of proteins with PrLDs in the proteomes of different organisms seems to differ significantly (Michelitsch and Weissman, 2000; Espinosa Angarica et al., 2013; Malinovska et al., 2015; Chakrabortee et al., 2016; Pallares et al., 2018), their presence in all evolutionary lineages analyzed so far suggests that these regions might play conserved functional roles (Michelitsch and Weissman, 2000; Malinovska et al., 2015; Batlle et al., 2017c).

**FIGURE 1:**
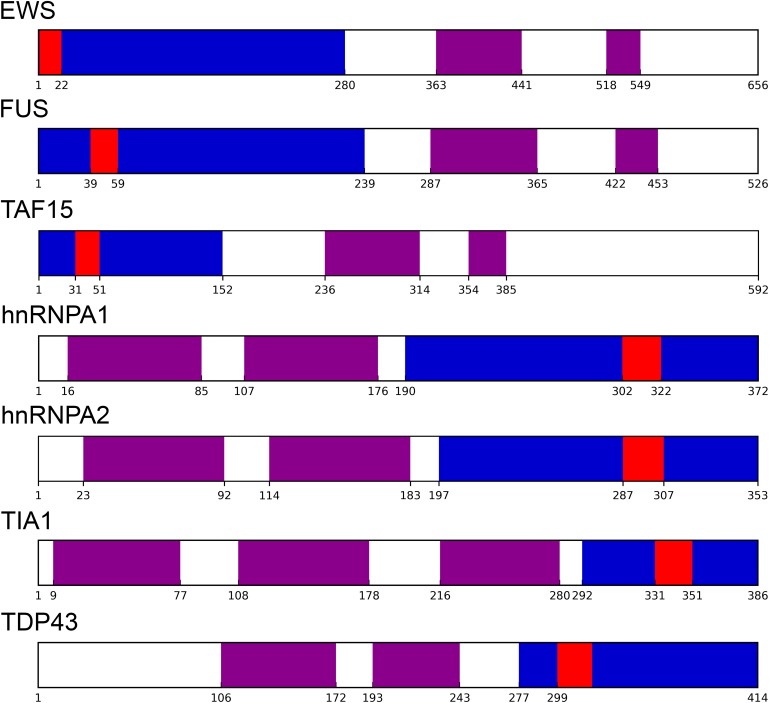
Prion-like proteins modularity. Well-characterized prion-like human proteins have their PrLD (as identified by PLAAC in blue) and soft amyloid core (as identified by pWALTZ in red) at the protein edges, separated from their respective globular domains (retrieved from Pfam database in violet).

**FIGURE 2:**
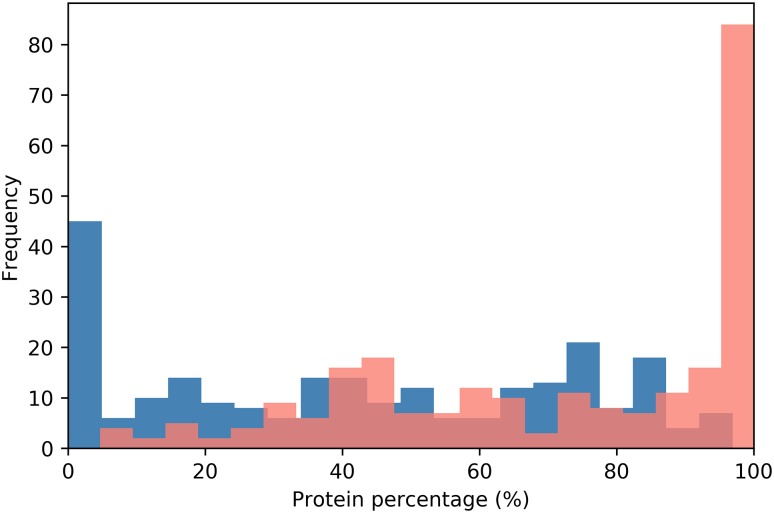
PrLD distribution along the protein sequence. The relative position of PrLDs in the sequences of the complete protein dataset is plotted. Protein sequences were divided into 20 bins corresponding to 5% of their length and the PrLDs start position represented in blue and the end in red.

Yeast prion proteins tend to be modular (Li and Lindquist, 2000; Alberti et al., 2009). PrDs being generally located near the N- or C-terminal ends of the sequence (Baxa et al., 2007; Zambrano et al., 2015). In our dataset, 195 proteins; an 80.6% of the putative human prion-like proteins, presented their PrLDs located in any of the protein’s ends (Figure 1, 2 and Supplementary Table S1). PrLDs were 1.67 times more frequent at the protein C-terminus. This was the case for 122 proteins, while in 73 of them the PrLDs were located at the N-terminus. This statistically significant imbalance between the presence of PrLDs at C- and N- in human proteins (*p*-value < 0.005, *Z*-test), contrasts with that found in *bona fide* yeast PrDs. In SUP35, URE2, NEW1, MOT3, and SWI1 proteins, the PrD is placed at the N-terminus, whereas only in RNQ1, it is located near the carboxyl end (Baxa et al., 2007; Zambrano et al., 2015). The modular architecture of prion-like proteins would allow the self-assembly of the PrLDs without disturbing the structure and productive associations of the adjacent globular moieties. This is likely facilitated by the predicted disordered nature of these protein segments (Supplementary Table S1).

### Human Prion-Like Proteins Play a Major Role in Nucleic Acid Binding

**FIGURE 3:**
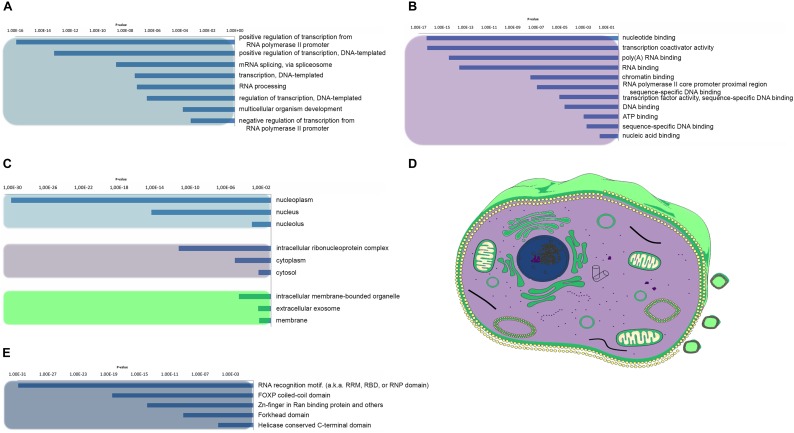
Human prion-like proteins GO enrichment analysis. The prion-like proteins GO enrichment was performed and separated into its three ontologies. **(A)** Biological process. **(B)** Molecular function. **(C,D)** Cellular component. Clusters were grouped by color and represented with the same color code in a mammalian cell in **D**. **(E)** Pfam structural domains enriched in prion-like proteins were computed against the human proteome background.

As a first step to gain insights into the biological role of the candidate human prion-like proteins, we used a GO term analysis. GO terms were collected for biological process, molecular function, and cellular component categories and their enrichment with respect to the human proteome calculated (Figure 3). When we analyzed the “biological process” category for the set of candidate proteins, we found a statistically significantly enriched cluster of GO terms related to RNA and DNA associated processes, including positive regulation of transcription from RNA polymerase II promoter (*p*-value < 1.20E-16, 30 proteins), positive regulation of transcription DNA-templated (*p*-value < 6.92E-14, 22 proteins), mRNA splicing (*p*-value < 2.27E-9, 13 proteins), transcription DNA-templated (*p*-value < 5.26E-8, 36 proteins), RNA processing (*p*-value < 7.5E-8, 10 proteins), and negative regulation of transcription from RNA polymerase II promoter (*p*-value < 6.28E-4, 11 proteins) (Figure 3A). This result is consistent with the observation that the prion-like subproteomes identified in organisms belonging to different taxonomic divisions are usually enriched in proteins associated to the regulation of the flux of genetic information in the cell (Iglesias et al., 2015; Pallares et al., 2018).

With respect to the “molecular function,” the most enriched GO terms are all involved in essential activities related with nucleic acid binding and transcription processes, such as transcription coactivator activity (*p*-value < 5.63E-17, 20 proteins), nucleotide binding (*p*-value < 4.96 E-17, 37 proteins), poly(A)RNA-binding (*p*-value < 3.94E-15, 30 proteins), RNA-binding (*p*-value < 2.99E-14, 31 proteins), chromatin binding (*p*-value < 3.34E-14, 14 proteins), transcription factor activity-sequence-specific DNA binding (*p*-value < 9.79E-6, 29 proteins), and ATP binding (*p*-value < 1.14E-4, 13 proteins) (Figure 3B). The conformational plasticity of PrLDs has been shown to be behind certain transcription factors ability to bind to many different targets and to play a role in the formation of chromatin regulatory complexes (Boulay et al., 2017; Kataoka and Mochizuki, 2017; Cho et al., 2018). Moreover, it is becoming increasingly clear that PrLDs are crucial for the formation of membraneless organelles, since they enable RNA-binding proteins (RBPs) to undergo liquid–liquid transition, confining their RNA cargos (Villarroya-Beltri et al., 2013; Wang et al., 2018).

When we analyzed the cellular components populated by our protein subset, the most enriched GO terms were the nucleoplasm, nucleus, and the intracellular ribonucleoprotein complex (Figure 3C,D). As expected, all these compartments correspond to locations were the binding between nucleic acids and proteins occur frequently. Of particular interest is the so-called ribonucleoprotein complex which includes cellular structures like the stress granules, or P-bodies, which are sites for mRNA decay as well as for mRNA storage and therefore act as important cell regulatory centers in determining levels of gene expression (Anderson et al., 2015). The RBPs associated to those membrane-less organelles are key determinants in the control of the organelle function and have been implicated not only in adaptation to stress but also in tumor biology and the pathogenesis of neurodegenerative, immunological, and infectious diseases (Loomis et al., 1990; Villarroya-Beltri et al., 2013; Anderson et al., 2015; Harrison and Shorter, 2017).

We extended our analysis to look for the role of the constituent functional domains in the collection of PrLDs containing proteins. In agreement with the above presented results, Pfam domain clustering rendered DNA/RNA binding as the most enriched functional group (Figure 3E). Among them, the canonical RNA recognition motif (RRM) is by far the most statistically enriched, with 14% of the detected proteins harboring an RRM. This observation is line with previous studies (King et al., 2012) and consistent with the fact that the RRM is the most abundant domain in RBPs, conserved from bacteria to higher eukaryotes (Reddy et al., 2015). This set of RRM-bearing prion-like proteins includes FUS, TDP-43, TIA1, or hnRNP A1, all involved in the formation of dynamic membraneless intracellular compartments and associated to disease (Cascarina and Ross, 2014; March et al., 2016; Wang et al., 2018).

The second most enriched domain in our data set is the FoxP coiled-coil (*p*-value < 2.95E-19, 10 proteins). It corresponds to a coiled–coil domain involved in the modulation of the dimeric associations of the forkhead box family of transcription factors FoxP. There are multiple lines of evidence suggesting the biological relevance of domain swapping in FoxP functionality being important not only for their function regulation but also linked to disease onset (Hafner-Bratkovic et al., 2011; Medina et al., 2016).

The other two enriched Pfam families include Zinc-fingers in Ran binding proteins (Zn_RanBP) (*p*-value < 1.14E-14, 9 proteins) and the Helicase conserved C-terminal domain (*p*-value < 2.47E-05, 7 proteins). Zinc Finger domains are a very versatile group of small protein domains which are evolutionary conserved. Interestingly, RBPs with PrLDs such as FUS or EWS accommodate in their structure a Zn_RanBP domain in close proximity to an RRM domain. The Helicase conserved C-terminal domain is found at the C-terminus of DEAD-box helicases. Helicases function in the separation of double-stranded RNA, DNA, and RNA/DNA structures in an energy-dependent manner and therefore it is clear their role in RNA metabolism. Interestingly, the first prion-like protein identified in bacteria corresponds to the transcription terminator Rho, a helicase that can undergo a prion-state that results in genome-wide changes at the transcriptome level, contributing to rapid bacterial adaptation to fluctuating environments (Pallares et al., 2015; Yuan and Hochschild, 2017). The multitasking transcriptional regulators DDX5 and DDX17 included in our dataset contain an helicase domain in their structure reported to be associated with cancer development and cell proliferation (Mazurek et al., 2012; Fuller-Pace, 2013).

### Prion-Like Proteins Are Widespread Among Tissues

**FIGURE 4:**
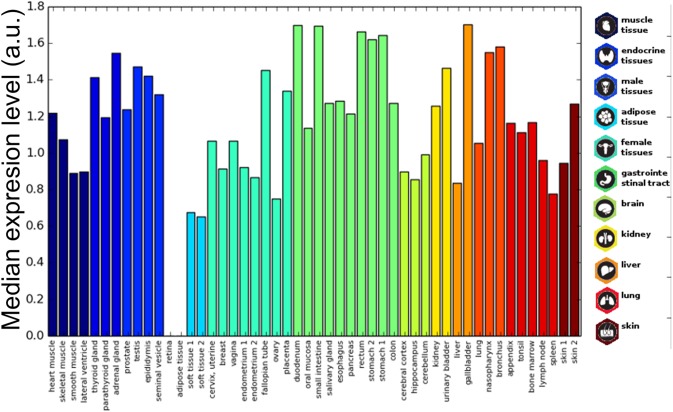
Prion-like proteins expression in tissues. The average expression of prion-like proteins dataset is plotted for different tissues. The tissue bars are colored based on the corresponding organ/tissue. Values range from 0 to 3 corresponding to Human Protein Atlas annotation “not detected,” “low,” “medium,” and “high.”

The histological localization of human prion-like proteins was assayed by retrieving data from the Human Protein Atlas. To compare the expression levels, proteins were mapped to Ensemble gene annotations (121 genes). The expression data were collected for each cell type and averaged by tissue and organ. The result illustrates that prion-like proteins are widely distributed in human tissues (Figure 4). Importantly, the data indicate that, globally, the expression of these proteins in the brain is not higher than in most organs or tissues, being more represented in endocrine tissues, in the gastrointestinal tract, the kidney, or the lung.

In order to identify interesting cases, we clustered the dataset by representing each gene as a vector of the difference of its expression with respect to the proteome-level tissue average (V_g_ = [ ( E - Ē )_1_ … ( E - Ē )*_*n*_* ] where V_g_ : the vector of gene expressions; E: gene expression in tissue *n*, and Ē: average expression of all human proteome in tissue n). The clustering was performed through k-means algorithm implementation of scikit-learn Python module, which uses Euclidean distances by default. We tested cluster numbers from 3 to 10 and chose 6 as the most discriminative one (Average silhouette, Supplementary Figure S1). Thus, the highest expression level cluster represents a group of prion-like proteins that are generally over-expressed and remarkably includes most of the human prion-like proteins for which it has been already demonstrated their direct involvement in disease: FUS, TDP-43, hnRNP A1, hnRNP A2/B1, hnRNP A3, hnRNP U, hnRNP H1, and EWS. Many of these proteins have already been described to be spread throughout most tissues and identified at different developmental stages (Bastian et al., 2008; Uhlen et al., 2015).

### Prion-Like Proteins Are Disease Related

**FIGURE 5:**
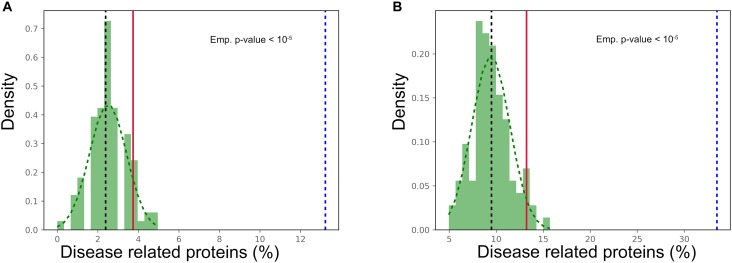
Prion-like proteins disease association. Number of disease associations for prion-like proteins (dotted blue line) compared to 100 random sampling of the human UniProt from **(A)** OMIM and **(B)** DisGeNET databases. The median of the background sample is plotted as a black dotted line, while the red line refers to the 95 percentile of the distribution (*p*-value < 0.05).

Given the widespread tissue distribution of the prion-like proteins and the link to disease of proteins in the most expressed cluster, we explored whether, globally, genes encoding for these polypeptides were connected to pathological processes. Their association to diseases was retrieved separately from the Online Mendelian Inheritance in Man (OMIM) (Amberger et al., 2015) and the database of gene-disease association (DisGeNET) (Pinero et al., 2017). The percentage of genes with disease annotations was calculated and compared with that in the complete human UniProt dataset, which was used as background. According to the OMIM database, 13.22% of the prion-like proteins encoding genes are disease-related against a 2.39% for the UniProt dataset, whereas values of 33.47 and 9.49% were obtained in the case of DisGeNET (*p*-value < 1.0E-5 for both databases, *Z*-test). Thus, the association with disease of prion-like proteins was threefold and fivefold higher than the one in the complete human proteome, according to DisGeNET and OMIM, respectively. To assess the significance of this enrichment, 100 random samples with the same size that the prion-like proteins dataset were selected from the background, the percentage of proteins associated to a disease in each sample was counted and the distribution of the percentages calculated (Figure 5). For both OMIM and DisGeNET, the prion-like dataset proportion is clearly above the 95 percentile of the distribution, which implies a significant over-representation of disease-associated proteins among human prion-like proteins. At this point, it is important to underline that the prion-like protein identification pipeline is sequence-based and totally blind with respect to the protein annotation.

Prion-like proteins have been associated to the onset of neurological disorders (Harrison and Shorter, 2017). The 9% of genes encoding for prion-like proteins, 11 out of 121, are linked to neurological diseases, according to OMIM (Supplementary Table S3). This constitutes a significant enrichment, relative to the complete proteome (*p*-value < 1.5 E-8). However, it is important to note that, despite proteins connected with neurological disorders are over enriched by 1.4-fold within the disease associated prion-like protein subgroup, this enrichment is not statistically significant (*p*-value > 0.11). It is clear from the results presented above that many of the detected proteins are ubiquitous regulators involved in a wide range of signaling pathways; which suggests that perturbations affecting their function may have a great impact in multiple disorders and not exclusively in neurological diseases, as it is usually assumed.

### Prion-Like Proteins’ Role in Highly Interconnected Subnetworks

**FIGURE 6:**
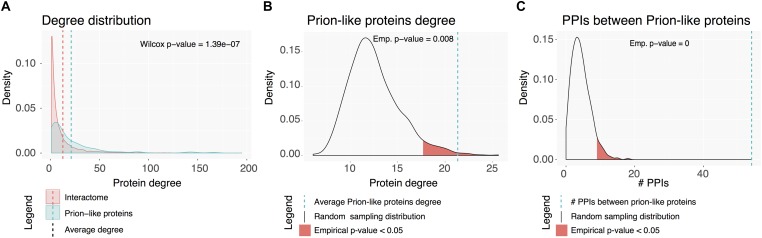
Human prion-like interactome. **(A)** Degree distribution for the complete interactome in red and the prion-like proteins network (first neighbors) in blue. **(B)** Prion-like proteins average interaction degree (blue dotted line) compared to a random sampling of 1000 sets. **(C)** Number of PPIs between prion-like proteins (blue dotted line) compared to a random sampling of 1000 sets.

Proteins rarely perform their functions independently; but mostly rely on complexes to carry them out. The connectivity of human prion-like proteins and the properties of their interactors were analyzed. As above, prion-like proteins were first mapped to genes to obtain unique entities. Out of the 121 resulting genes, 100 had annotated physical binary interactions (physical interactions between two individual proteins). Overall, prion-like dataset and the proteins they interact with establish a subnetwork of 1544 proteins with 2079 PPIs between them. Both the prion-like dataset and the complete subnetwork have higher average interaction degrees than the human interactome (Figure 6A). To uncover whether prion-like proteins interact more than expected by chance, the average degree of interactions of the prion-like protein set was compared with 1000 random sets of proteins of the same size (Figure 6B). This analysis confirms that prion-like proteins exhibit a significant higher number of interactions than the average human interactome. Next, we assessed whether prion-like proteins interact more between them than expected by chance, by comparing the number of intra-set interactions with that in 1000 random sets, as before. The results showed that prion-like proteins establish more interactions – one order of magnitude higher – between them than expected randomly (Figure 6C). To further describe the human prion-like subnetwork, it was tested to what extent prion-like proteins cluster into specialized interactome neighborhoods. The size of the LCC and the MSD was measured and compared to 1000 random sets (Table 1). The results clearly show that prion-like proteins share a higher interactomic vicinity than expected randomly, providing support to the concept that they exist well-defined interaction networks for human prion-like proteins.

**Table 1:** Prion-like proteins are located nearer in the network than expected by chance.

	Prion-like	Random
	protein set	expectation	*Z*-score	*P*-value
LCC size	32	3.16	16.8	<1.10^-5^
MSD	1.64	2.2	-6.78	<1.10^-5^


To functionally characterize this subnetwork of prion-like proteins and their interactors, the 1544 proteins were analyzed for GO and KEGG pathways enrichment. GO enrichment analysis are consistent with the results obtained for the prion-like proteins dataset alone, as it highlights regulation of gene expression through DNA and RNA binding as the main biological role played by this protein subset (Supplementary Table S4).

When we examined the statistically enriched pathways obtained from KEGG analysis, we observed that they can be grouped into two main clusters. Remarkably, the largest cluster collects pathways involved in different types of cancer, such as transcriptional misregulation in cancer (*p*-value < 9.86E-15, 53 proteins), pancreatic cancer (*p*-value < 2.01E-12, 29 proteins), prostate cancer (*p*-value < 1.31E-11, 33 proteins), or colorectal cancer (*p*-value < 1.88E-7, 22 proteins) among others; 12 prion-like proteins (10% of the total unique entries) and 122 (8.4%) of their interactors were found in these cancer related pathways. These interactors include cornerstones in mitogenesis, growth factor signaling, apoptotic attenuation, cell cycle progression, angiogenesis, cell invasion, immune regulation, and microenvironment alterations.

The second group encompasses pathways associated with viral infection, such as viral carcinogenesis (*p*-value < 1.42E-15, 61 proteins), Epstein–Barr virus infection (*p*-value < 3.91E-14, 56 proteins), herpes simplex infection (*p*-value < 6.6E-12, 51 proteins), or hepatitis C (*p*-value < 1.07E-6, 38 proteins). This is consistent with the involvement of RBPs, helicases, and splicing-related proteins in the control of viral assembly and trafficking of the viral genomic RNA from the nucleus. Prion-like candidates such as DDX17 (Moy et al., 2014), DDX5 (Cheng et al., 2018), and hnRNP A2B2 (Levesque et al., 2006) have been already described to play key roles in these processes.

## Discussion

In the present work, we used a stringent computational approach that considers that PrLDs should not be only disordered and compositionally biased, but also encode for short sequences with moderate, but significant, amyloid propensity (Sabate et al., 2015b). We concluded that 242 polypeptides in the human proteome fulfill the requirements to potentially behave as prion-like proteins. This accounts for less than 1% of the human proteins, which implies that, compared with organisms like *Plasmodium* or *Dictyostelium* where 10–25% of their proteins are predicted be prionogenic (Singh et al., 2004), the prionic load of the human proteome is low. The dataset included several widely studied proteins with prion-like behavior, such as FUS, TIA1, TDP-43, EWS, and several hnRNPs, but also previously undescribed proteins with very important cellular functions: members of the mediator complex, nucleoporins, chromatin remodeling proteins, and transcription factors.

As their counterparts in yeast (Santoso et al., 2000; Alberti et al., 2009), human prion-like proteins, locate their PrLDs mostly at their ends; with a slight preference for the amino terminus. This might imply that the position of the PrLD within the protein sequence might be relevant for its function. Indeed, previous analyses on proteins containing low complexity regions already suggested that these terminal positions would allow them to act as act as promiscuous interfaces for protein binding, without steric interferences by the adjacent globular domains (Coletta et al., 2010). In a similar manner, prion-like modularity and the preference for terminal regions are likely maintained in order to delimit a flexible region which can switch its conformation and assemble, modulating in this way the activity of folded domains without impacting their native 3D structure.

According to the GO terms analysis, a highly significant fraction of prion-like proteins are involved in functions related to nucleic acid binding and transcription and translation activities. This includes proteins of the Mediator complex, implicated in the regulated transcription of nearly all RNA polymerase II-dependent genes (Zhu et al., 2015; Cho et al., 2018), proteins recruited in chromatin-remodeling complexes (Boulay et al., 2017; Kataoka and Mochizuki, 2017), and a significant number of transcription factors. The dataset also includes the large majority of RBPs already described to behave as prion-like in humans, such as FUS which is implicated in transcription, DNA repair, and RNA biogenesis (Patel et al., 2015), TIA1 which functions in mRNA turnover and regulation of translation (Li et al., 2014), TDP-43 which is involved in transcriptional regulation and RNA processing (Buratti and Baralle, 2008; King et al., 2012), EWS which is implicated in RNA binding and processing, or diverse hnRNPs involved in the packaging of pre-mRNA into RNP particles (He and Smith, 2009). Not surprisingly, we found that a high proportion of these proteins map into the nucleus and intracellular ribonucleoprotein complex. This last observation is consistent the extensive literature identifying prion-like sequences as drivers of liquid–liquid phase separation in membrane-less cellular compartments (Patel et al., 2015; Banani et al., 2017).

Our data reveal that human prion-like proteins are multifunctional proteins involved in important regulatory processes. Indeed, 50% of the proteins in our dataset carry at least two different Pfam domains. As expected from the molecular functions in which these proteins are involved, the most statistically enriched domains correspond to RNA and DNA binding domains such as the canonical RRM, the Zn finger domain, the forkhead domain, or the helicase domain. All of them present in well-characterized transcription factors and RNPs. These are evolutionary conserved domains in which, because of their functional relevance, genetic mutations are often linked to disease (King et al., 2012; Cascarina and Ross, 2014).

We assessed the expression of genes coding for prion-like proteins for each human tissue, to try to rationalize why, so far, these proteins have been mostly related to neurological diseases. Human prion-like protein expression was not restricted to nervous tissue but ubiquitously spread among tissues; also, they are not especially abundant in the brain, relative to other organs of the human body. This suggests that they play a physiological role in different cellular types, although it raises the question of why most prion-like proteins related diseases are tissue-specific. This situation is not unique for prion-like proteins but common to other proteins involved in neurodegenerative disorders, i.e., a-synuclein the protein responsible for Parkinson’s disease, is abundantly expressed in both the cerebral cortex and the bone marrow, but only aggregates in the brain (Spillantini et al., 1997; Barbour et al., 2008). The protein quality control machinery has an active role in managing protein misfolding and aggregation. Cellular aging impacts cell homeostasis and leads to proteostatic-compromised cells in which misfolding and aggregation events cannot be compensated (Aguzzi and Altmeyer, 2016). It has been proposed that the low efficacy of replacing dying neurons, relative to other cells types, could be one of the underlying reasons why the malfunction of prion-like proteins is more often associated to neurological conditions. One important finding here is that many of the human prion-like proteins that have been convincingly associated to disease are among the most expressed polypeptides in the dataset. This fits very well with the so-called “life at the edge” hypothesis, which states that, because protein aggregation is extremely dependent on concentration, abundant proteins are, on the average, at highest risk of misfolding and aggregation (Tartaglia et al., 2007).

Independently of their tissue distribution, what becomes clear from the analysis of the OMIM and DisGeNet databases is that human prion-like proteins are strongly connected to disease. Two complementary properties might explain, at least in part, this strong association. First, the propensity of PrLDs to establish intermolecular interactions together with the presence of regions with significant amyloid propensity, exposed to solvent within large disordered regions, impose an inherent risk to aggregate to these polypeptides. In fact, genetic mutations that increase the aggregation propensity of PrLDs have been shown to be directly associated with disease (Harrison and Shorter, 2017). Second, according to the “centrality-lethality rule” (Jeong et al., 2001), the highest the number of interactions for a protein is, the largest is the impact of its disruption on cell function. Thus, the high connectivity of prion-like proteins networks might well account for their strong link to human diseases. Importantly, KEGG pathway enrichment analysis of the prion-like proteins interactome allowed us to uncover a highly significant association with two previously undescribed set of devastating pathological processes: cancer and viral infections.

Overall, despite the present study constitutes only a first theoretical approach to the function of human prion-like proteins, our results indicate that this subproteome exert important regulatory functions in different biological pathways, thanks to both their protein-protein and protein-nucleic acids binding capabilities, two properties that seem to be favored by their modular architecture. The analysis suggests that in the forthcoming years, we can expect the discovery of a connection between prion-like proteins malfunction and other pathologies apart from neurological disorders.

## Data Availability

All datasets generated for this study are included in the manuscript and/or the Supplementary Files.

## Author Contributions

IP, PA, ST, and SV conceived the experiments and analyzed the results. VI, LP, and TJ-B conducted the experiments and prepared the figures. VI, IP, and SV wrote the main manuscript text. All authors reviewed the manuscript.

## Conflict of Interest Statement

The authors declare that the research was conducted in the absence of any commercial or financial relationships that could be construed as a potential conflict of interest.

## Abbreviations

GO: gene ontology
LCC: largest connected component
MSC: mean shortest distance
PPIs: protein–protein interactions
PrD: prion domain
PrLD: prion-like domain
