# Supplementary material for: In silico Characterization of Human Prion-Like Proteins: Beyond Neurological Diseases
Source: Front Physiol. 2019 Mar 27;10:314. doi: 10.3389/fphys.2019.00314 (PMC6445884; doi:10.3389/fphys.2019.00314)
Supplement: Supplementary file 3 [file Table_3.DOCX]

| Ensembl gene ID | Uniprot accession number | Name | Annotated diseases (as retrieved from OMIM) |
| --- | --- | --- | --- |
| ENSG00000112592 | P20226 | TBP, TATA box binding protein | Spinocerebellar ataxia 17. |
| ENSG00000085224 | P46100 | ATRX, Alpha thalassemia/mental retardtn. syndrome X-linked | Mental retardation-hypotonic facies syndrome, X-linked. |
| ENSG00000089280 | P35637 | FUS RNA binding protein | Amyotrophic lateral sclerosis 6, with or without frontotemporal dementia.  Tremor, hereditary essential, 4. |
| ENSG00000182944 | Q01844 | EWSR1, EWS RNA-binding protein 1 | Neuroepithelioma. |
| ENSG00000111752 | P78364 | PHC1, Polyhomeotic homolog 1 | Microcephaly 11, primary, autosomal recessive. |
| ENSG00000116001 | P31483 | TIA1, TIA1 cytotoxic granule-associated RNA binding protein | Welander distal myopathy. |
| ENSG00000114354 | Q92734 | TFG, TRK-fused gene | Hereditary motor and sensory neuropathy, Okinawa type.  Spastic paraplegia 57, autosomal recessive. |
| ENSG00000120948 | Q13148 | TARDBP, TAR DNA binding protein | Amyotrophic lateral sclerosis 10, with or without FTD.  Frontotemporal lobar degeneration, TARDBP-related. |
| ENSG00000122566 | P22626 | HNRNPA2B1, Heterogen. nuclear ribonucleoprotein A2B1d | Inclusion body myopathy with early-onset Paget disease. |
| ENSG00000135486 | P09651 | HNRNPA1, Heterogeneous nuclear ribonucleoprotein A1d | Amyotrophic lateral sclerosis 20.  Inclusion body myopathy wtih early-onset Paget disease without frontotemporal dementia 3. |
| ENSG00000152795 | O14979 | HNRNPDL, Heterogeneous nuclear ribonucleoprotein D-like | Limb-girdle muscular dystrophy, type 1G. |

**Table S3.** Identified prion-like genes linked to neurodegenerative conditions in humans. Diseases associations were retrieved from OMIM (Amberger et al., 2015).
