# Supplementary material for: In silico Characterization of Human Prion-Like Proteins: Beyond Neurological Diseases
Source: Front Physiol. 2019 Mar 27;10:314. doi: 10.3389/fphys.2019.00314 (PMC6445884; doi:10.3389/fphys.2019.00314)
Supplement: Supplementary file 5 [file Image_1.pdf]

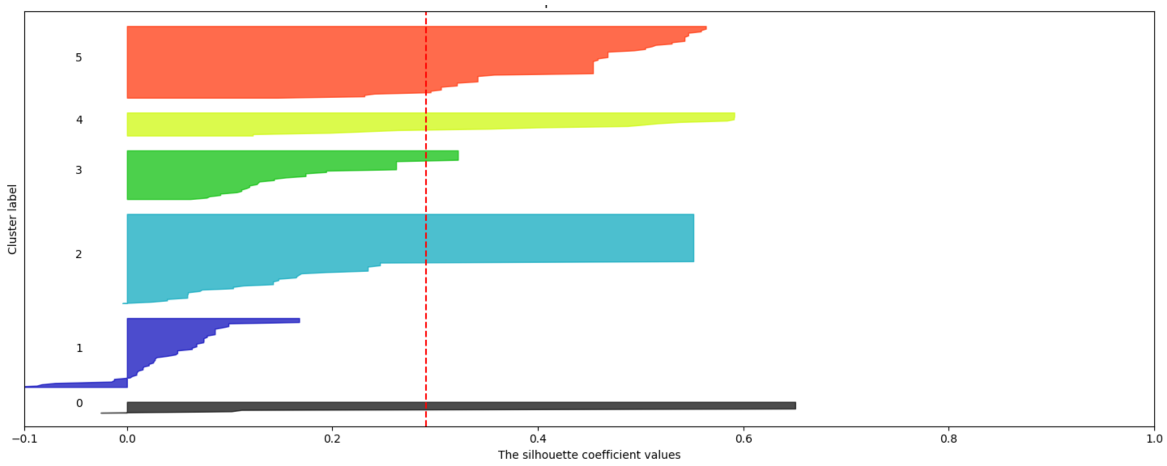


**Figure 1. Silhouette analysis for KMeans clustering on sample data with number of clusters = 6.** The average silhouette analysis was performed through the metrics implementation of scikit-learn Python module, for number of clusters between 3 and 10. All the silhouette scores range from 0.28 to 0.32. We aimed at the identification of a group of genes with general higher expression compared to the others. Therefore, in addition to the average silhouette plots, we took into account the size of best scoring cluster in order to end up with a number of genes for which it was possible to manually curate data (such as the disease association). n_clusters = 3 was discarded because output clusters were too large to do that. From n_clusters = 4 onwards, all plots contain at least one cluster with below average silhouette score and possibly misclassified examples. We excluded n_clusters = 7, 8, 9 and 10 as they contained more than one of them. Among n_clusters = 4, 5 and 6 we selected 6 as it contains the clusters with more similar sizes.
